# Evolution of Knowledge in the Treatment of Long-Standing Atrial Fibrillation in a UK Tennis Champion

**DOI:** 10.7759/cureus.14624

**Published:** 2021-04-22

**Authors:** Shiv Krishnaswamy, Manas Rane, J. Michael Gaziano, Charles Hennekens

**Affiliations:** 1 Cardiology, Charles E. Schmidt College of Medicine, Boca Raton, USA; 2 Cardiology, VA Boston Healthcare System, Harvard Medical School, and Brigham and Women’s Hospital, Boston, USA

**Keywords:** atrial fibrillation, rate control, digoxin, beta adrenergic blockers, stroke prophylaxis, aspirin, apixaban

## Abstract

During the last several decades, there have been major advances in the evolution of drug therapies for the rate management of atrial fibrillation (AF). Initially, the drug of choice was digoxin but currently, the drug of choice is beta-adrenergic blockers. Drug therapies for stroke prevention in AF have also evolved. Initially, the drug of choice was aspirin, then became warfarin, and now in the current era, there are newer oral anticoagulants, such as apixaban, which are the preferred drugs. In this case report, we present the details of a 79-year-old athletic man who developed palpitations due to rapid AF at age 31. At the time of his initial presentation, he was treated with digoxin and aspirin and has remained on these drugs to the present. In 1973, 28 years after his initial presentation, he became the United Kingdom (UK) amateur tennis champion in the 55 and over division at age 59.

At present, the clinical applications of advances in the management of AF should include quality of life considerations in the context of patient preferences. This patient is an active and vigorous 79-year-old man who plays competitive tennis and pickleball. He steadfastly adheres to an antediluvian regimen for the management of his AF, but this may be viewed in the context of the famous quotation by Bert Lance, Director of the Office of Management and Budget in the US under President Carter who said “sometimes, if it ain’t broke, don’t fix it.”

In addition to the evolution of drug therapies from digoxin to beta-adrenergic blockers for rate control as well as from aspirin to warfarin to apixaban for the prevention of stroke, there have been other recent remarkable advances. For example, recent promising findings from randomized trials include that early rhythm control was more effective than rate control as well as that cryoballoon ablation was superior to drug therapies. These findings require confirmation in additional randomized trials designed a priori to test these promising but unproven hypotheses.

## Introduction

Atrial fibrillation (AF) is the most common cardiac arrhythmia lasting greater than 30 seconds. In the United States (US), AF affects approximately 4% of adults over the age of 60. In addition, the incidence, severity and mortality from untreated AF increase with age. Based on the aging of the US population, it has been estimated that AF will increases 2.5-fold within the next 50 years [1]. AF has been classified as long-standing, paroxysmal, persistent, and permanent [2]. The risk factors that contribute to increased mortality from AF include, but are not limited to, obesity, diabetes mellitus, cerebrovascular disease, chronic kidney disease, and cancer. During the last several decades, there have been major advances in the evolution of drug therapies for AF. These include rate management, which has shifted from digoxin to beta-adrenergic blockers as well as stroke prevention from aspirin to warfarin to apixaban. In this case report, we present a unique history of a 79-year-old athletic man who developed palpitations due to rapid AF at age 31. At the time of his initial presentation, he was treated with digoxin and aspirin and has remained on these drugs to the present. In 1973, 28 years after his initial presentation, he became the United Kingdom (UK) amateur tennis champion in the 55 and over division at age 59. 

We discuss the signs, symptoms, diagnosis and treatment of his AF in the context of the evolution of newer and more efficacious drug therapies for rate management as well as stroke prevention. The timeline is from 1973 to the present.

## Case presentation

**Figure 1:**
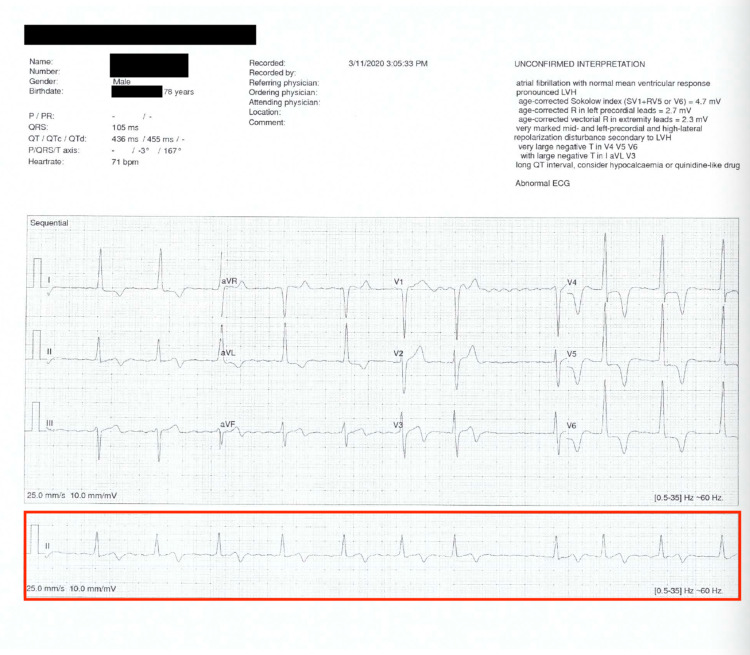
Patient's EKG from 2020 showing atrial fibrillation with VR of 72.

The patient (AS) is a 79-year-old Caucasian man who was born and raised in London, United Kingdom (UK). As a lifelong conditioned athlete, his resting heart rate had been 40 to 50 beats per minute (bpm) in normal sinus rhythm until 1973. After competing in and winning a tennis tournament, he presented to his general practitioner with palpitations but had no shortness of breath, weakness, tiredness, reduced ability to be physically active, lightheadedness or dizziness. His electrocardiogram (ECG) showed AF with a ventricular rate (VR) of 120. He was digitalized and maintained on 0.125 milligrams (mg) digoxin daily. He refused further medical follow-up and declined to take warfarin due to his perception of an increased risk of bleeding, but chose 325 mg aspirin daily. In 1999, at age 59 he won the UK national amateur tennis championship in the 55 and over division. In 2002, he moved to Boca Raton, Florida, US but returned to the UK for hip replacements in 2007 and 2017. He lives at home with his wife and remains physically active walking daily as well as playing tennis and pickleball three to five times per week. A few months ago, he went for a routine physical examination which revealed an alert, lean and fit man looking younger than his stated age with an oxygen saturation of 98% on room air in no respiratory distress. He had no jugular venous distention. His pulse was irregularly irregular, and ECG showed AF with a VR of 72 (Figure 1). 

His blood pressure was 140/90 mm Hg. Cardiac exam revealed no murmurs and he had no lower extremity edema.

His Congestive Heart Failure, Hypertension, Age, Diabetes mellitus, Stroke-Vascular Disease, Age, Sex Category (CHADS-VASC) score was three, which predicted an annual risk of stroke of 3.2% per year [3,4]. His Hypertension, Abnormal Renal and Liver Function, Stroke, Bleeding, Labile International Normalized Ratio, Elderly, Drugs or Alcohol (HAS-BLED) score was two, which predicted an annual risk of bleeding of 4.1% [5].

The patient was advised to discontinue digoxin and monitor his VR which has ranged from 44 to 74 beats per minute. He was prescribed apixaban but refused and elected to remain on aspirin. Since then he has restarted digoxin and his resting VR ranges from about 44 to 74.

## Discussion

With respect to the management of his rapid AF, at the time of his initial presentation with palpitations due to rapid AF, digoxin was commonly used to control the VR. Digoxin was first isolated in 1930 from the foxglove plant, Digitalis lanata, and currently holds a place on the World Health Organization’s List of Essential Medicines [6,7]. Since that time the evolution of medical knowledge has resulted in guidelines from the European Society of Cardiology recommending beta-adrenergic blockers as the first-line drug of choice for rate control in rapid AF [8]. Historically, the initial trials of beta-blockers were small in sample size and the majority of patients were already taking digoxin. Subsequent trials, however, showed an optimal benefit to risk ratio of beta-blockers alone [9,10]. This patient has refused this option, based in part, on his perceptions of deleterious impacts of beta-blockers on strenuous exercise as well as erectile function [11].

Previous randomized trials directly testing rate versus rhythm control in AF showed no significant differences in mortality between the two treatment strategies [12-14]. In fact, rate control therapy is still the most widely used therapeutic modality in patients with AF due to the lower risks of adverse effects. In a recently reported trial, however, rhythm control was superior to rate control in patients newly diagnosed with AF. Specifically, The Early Treatment of Atrial Fibrillation for Stroke Prevention Trial (EAST 4AFNET) was a randomized trial designed to test whether rhythm control was superior to usual care in patients diagnosed with AF within one year [15]. In this trial 2,789 patients aged 75 years or older were randomized. The primary outcome was a composite of death from cardiovascular causes, stroke, or hospitalization with worsening heart failure or acute coronary syndrome. The trial was stopped early, based on the recommendation of the Data Monitoring Committee, due to the emergence of a statistically extreme effect on the primary outcome. At the time of early termination, after a median follow-up of 5.1 years, a first primary outcome event had occurred in 294 patients assigned to rhythm control (3.9 per 100 person years) and in 316 assigned to usual care (5.0 per 100 person years) (hazard ratio (HR), 0.79; 95% confidence interval (CI) 0.66 to 0.94; p-value = 0.005). In addition, there was no significant difference in the primary safety outcome (composite of death from any cause, stroke, or prespecified serious adverse events showing complications of rhythm control therapy) in the two treatment arms (early rhythm control, 16.6%; usual care 16.0%) [15]. This trial included ablation in the rhythm control group which was initiated within one year. Another recent trial, the Cryoballoon Catheter Ablation in an Antiarrhythmic Drug Naïve Paroxysmal Atrial Fibrillation (STOP AF First) trial tested drug therapy versus cryoballoon ablation in patients with paroxysmal AF. This trial randomized 203 patients aged 18 to 80 years. In the cryoballoon ablation group, treatment success at 12 months was 74.6% (95% CI, 65.0 to 82.0) while in the drug therapy group treatment success was 45% (95% CI, 34.6 to 54.7) (p < 0.001) [16]. While the current guidelines recommend rate control in asymptomatic patients with AF, and antiarrhythmic drugs to be used before considering ablation, the results of these two recent trials may signal a sea change for the management of AF. Specifically, they suggest the possibility to consider early rhythm control, and possibly ablation as first-line therapeutic strategies. At present, further randomized evidence is necessary to confirm or refute these promising but unproven findings. 

As regards stroke prevention, this patient has refused to follow the advice of his healthcare providers when warfarin was the preferred alternative and most recently apixaban. Instead, he has elected to take aspirin uninterrupted since 1973. It is interesting to note that our retrospective calculation of his CHADS-VASC score based on his data at presentation in 1973 was 0. By 1991, a major advance in medical knowledge occurred following the publication of the Stroke Prevention in Atrial Fibrillation (SPAF) trial [17]. The SPAF trial directly compared benefits and risks of warfarin, aspirin, and placebo for stroke prevention in patients with nonvalvular AF. 1,330 patients were stratified by eligibility for warfarin: Warfarin-eligible patients (Group 1) were randomized to warfarin (goal international normalized ratio of 2.0-4.5), aspirin 325 mg/day, or placebo, while warfarin-ineligible patients (Group 2) were randomized to aspirin 325 mg/day or placebo. At a mean follow-up of 1.3 years, the rate of ischemic stroke or systemic embolism was significantly reduced in patients assigned at random to warfarin compared with those assigned placebo (2.3% vs. 7.4% per year), yielding a statistically significant and clinically important relative risk reduction of 67%. With respect to the aspirin and placebo comparisons, primary events were significantly less frequent in the aspirin group (3.6% vs. 6.3% per year) yielding a 42% relative risk reduction. Major complications, chiefly major bleeding, were 1%-2% per year in each group and not significantly different. Intracranial bleeding was rare and similar among patients assigned a random to warfarin (n = 2), aspirin (n = 2), or placebo (n = 2). Following SPAF and subsequent trials, warfarin became the standard of care for stroke prevention in eligible patients with nonvalvular AF. In patients at lower risk of stroke or higher risk of bleeding, aspirin was still often utilized despite lower efficacy.

In 2011, the Apixaban Versus Acetylsalicylic Acid to Prevent Stroke in Atrial Fibrillation Patients Who Have Failed or Are Unsuitable for Vitamin K Antagonist Treatment (AVERROES) trial was another major advance in medical knowledge. This randomized trial directly compared the benefits and risks of apixaban and aspirin on the primary prespecified endpoint of stroke or embolism. This trial was terminated early, based on the unanimous recommendation of the external and independent Data Monitoring Committee, after median of 2.8 years of treatment and follow up due to the emergence of a statistically extreme 55% benefit with apixaban (HR, 0.45; 95% CI 0.32 to 0.62; p < 0.001) and a possible but nonsignificant 30% (HR, 1.13; 95% CI 0.74 to 1.75; p = 0.57) higher bleeding risk [18]. With respect to early termination for efficacy, the results are reliable on the primary prespecified endpoint but less reliable as well as less robust data on other outcomes. We believe that the totality of evidence on apixaban versus aspirin, including AVERROES, indicates a clear and conclusive reduction in the risk of stroke with a small but significant increase in major bleeding. Although apixaban has been strongly recommended for this patient he steadfastly refuses to follow the advice of his healthcare providers. His major clinical concerns relate to major bleeding, especially head trauma due to his strenuous daily exercise routine. His predicted annual risk of embolic stroke is 3.2% based on the CHADS-VASC score [3,4] and his risk of a significant bleed is 4.1% based on the HAS-BLED score [5]. Thus, apixaban would have reduced his absolute risk of stroke from 3.2% to 1.6% with small increases in major bleeding when compared with aspirin whose predicted reduction is from 3.2% to 2.4%.

## Conclusions

The clinical applications of advances in the management of AF should include quality of life considerations in the context of patient preferences. This patient is an active and healthy 79-year-old man. Based on many competitive tennis and pickleball matches between several coauthors and this patient, we can attest that he has the speed and stamina of someone half his age. Thus, we conclude that while he steadfastly adheres to an antediluvian regimen for the management of his AF, we are also cognizant of the famous quotation by Bert Lance, Director of the Office of Management and Budget in the US under President Carter who said “sometimes, if it ain’t broke, don’t fix it.”

In addition to the evolution of drug therapies from digoxin to beta-adrenergic blockers for rate control and from aspirin to apixaban for the prevention of stroke, there have been other recent remarkable advances. For example, the recent promising findings that, early rhythm control was more effective than rate control as well as that cryoballoon ablation was superior to drug therapies require confirmation in additional randomized trials designed a priori to test the hypotheses. Therefore, the optimal prescription by health care providers and utilization by patients of all these modalities are likely to further reduce avoidable premature morbidity and mortality due to AF.
